# Plant establishment on unirrigated green roof modules in a subtropical climate

**DOI:** 10.1093/aobpla/pls049

**Published:** 2012-12-20

**Authors:** Bruce D. Dvorak, Astrid Volder

**Affiliations:** 1 Department of Landscape Architecture and Urban Planning, Texas A&M University, College Station, TX 77843, USA; 2 Department of Horticultural Sciences, Texas A&M University, College Station, TX 77840, USA

**Keywords:** Conservation, drought tolerant, establishment, extensive green roof, irrigation.

## Abstract

**Background and aims:**

The application of green roof technology has become more common in the central, northwestern and eastern USA, and is now being employed across the southern USA as well. However, there is little research in the literature that evaluated plant survival on unirrigated green roofs in subtropical climates that experience frequent drought and heat stress. Here, we summarize the results of a study of plant establishment on a modular green roof in south-central Texas.

**Methodology:**

Fifteen plant species were field tested in 11.4-cm-deep green roof modules on a four-storey building in College Station, Texas, with irrigation limited to the first several weeks of establishment. Climate data, plant growth and species survival were measured over three growing seasons.

**Principal results:**

Four species survived growing seasons without any losses: *Graptopetalum paraguayense*, *Malephora lutea*, *Manfreda maculosa* and *Phemeranthus calycinus*. Six species experienced varying levels of mortality: *Bulbine frutescens*, *Delosperma cooperi*, *Lampranthus spectabilis*, *Sedum kamtschaticum*, *Sedum mexicanum* and *Nassella tenuissima*. Five species had no survivors: *Dichondra argentea*, *Stemodia lanata*, *Myoporum parvifolium*, *Sedum moranense* and *Sedum tetractinum*.

**Conclusions:**

The establishment and survival of several plant species without any mortality suggests that irrigation limited to the first few weeks after planting may be an effective approach on green roofs in spite of the more challenging climatic conditions in the southern USA. Since the climate in south-central Texas had been consistently drier and warmer than normal during the study period, longer-term research on these species is recommended to expand knowledge of establishment requirements for these species under a wider range of conditions, including wetter than normal years.

## Abstract (precis)

Shallow-rooted plants were studied on unirrigated modular green roof trays. Four species had 100% survival, six had varied survival rates and five had no survivors. These outcomes suggest that water conservation practices can be an effective approach for green roofs.

## Introduction

The use of green roofs as a self-reliant and low-input technology emerged in Europe, Scandinavia and the UK, where climate conditions are favourable for maintaining green roof vegetation without irrigation. Criteria for selecting vegetation for unirrigated green roof systems are articulated in the ‘Guidelines for the Planning, Construction and Maintenance of Green Roofing’, commonly referred to as the German FLL Guidelines for green roofs (FLL 2008). The guidelines state, ‘green roofs are designed to depend primarily on precipitation for their water supply’ (p. 47), and thus the guidelines define performance expectations for substrate depth, composition and stability, porosity, nutrient- and water-holding capacity as well as plant watering requirements.

In Europe, hundreds of plant species have been identified for use on green roofs (Cantor 2008). As green roof technology continues to emerge beyond the climates of Europe, more information on the performance of a range of plant species on green roofs is needed. For example, in the semi-arid regions of Australia, the lack of data regarding plants for green roofs is a barrier to the growth of the green roof industry (Williams *et al*. 2010). In central Taiwan, 31 species of green roof plants were grown in planting dishes to test drought tolerance and seven species demonstrated normal growth (Liu *et al*. 2012). In North American climate zones that are much drier and warmer than Europe (Kottek *et al*. 2006), little is known about viable plant species on green roofs. In a recent review of North American green roof vegetation research, 40 succulent species and 94 herbaceous species were identified on green roofs across 15 ecoregions (Dvorak and Volder 2010). Only a few species of plants have been found to support vegetation on shallow unirrigated green roofs in climates that frequently experience heat stress and drought (Dvorak and Volder 2010; Sutton *et al*. 2012).

Extensive-type green roofs are typically shallow (<12 cm) and are dominated by succulents, whereas simple-intensive green roofs are typically deeper (12–35 cm) and can accommodate forbs and grasses (Köehler 2003, 2007; Dunnett and Kingsbury 2004; Dunnett and Nagase 2007; Sutton *et al*. 2012). Succulents are a popular choice for shallow unirrigated green roofs because of their ability to tolerate well-drained soil, drought conditions and shallow substrate depths (Earth Pledge, 2005; Snodgrass and Snodgrass 2006; Cantor 2008). Many succulents found to be successful in Europe have also performed well on green roofs in North America in the Upper Midwest (Durhman *et al*. 2006; Rowe *et al*. 2012), the Pacific Northwest (Hauth and Liptan 2003), New England and Nova Scotia (Lundholm *et al*. 2010; MacIvor and Lundholm 2011; Barker and Lubell 2012). With the growth of the green roof market in the southern USA, it is important to understand which plant species are viable on green roofs in southern climates with or without irrigation.

The purpose of this research was to identify species and their survival on unirrigated green roofs in a subtropical climate that is characterized by hot and dry summers interspersed with large precipitation events. We selected 15 species that were known for their drought tolerance as well as their ability to withstand the occasional winter freeze. In addition, these species were chosen for their ability to maintain their root system in shallow, well-drained, soils. We hypothesized that all of these species would show good survival without supplemental irrigation after an initial irrigated establishment period.

## Methods

### Study site characteristics

The research site was located in College Station, Texas (30°37′N, 96°20′W), which lies south and east of the geographic centre of Texas in a humid subtropical climate (Larkin and Bomar 1983) at ∼150 m elevation. College Station typically experiences daytime maximum temperatures >32.0 °C over 100 days a year. College Station's mean annual precipitation is 1011.2 mm, although only 233.9 mm falls between June and the end of August, which coincides with high diurnal temperatures (Table 1).

**Table 1:** **The College Station, Texas, 30-year climate characteristics (bold) compared with monthly means from 2009–2012.** Monthly means and annual means are presented for maximum, minimum and mean temperatures (°C), and precipitation (mm). Arrows indicate 10 % or greater deviation from long-term means.

	Jan	Feb	March	April	May	June	July	Aug	Sep	Oct	Nov	Dec	Annual
**Mean temp. (°C)**	**10.45**	**12.40**	**16.18**	**20.18**	**24.46**	**27.74**	**29.19**	**29.47**	**26.52**	**21.46**	**15.90**	**11.18**	**20.46**
**Mean max. temp. (°C)**	**16.17**	**18.22**	**22.11**	**26.11**	**29.89**	**33.22**	**34.94**	**35.61**	**32.56**	**27.50**	**21.72**	**16.94**	**26.3**
2009	—	—	—	25.67	30.56	36.39	38.17	37.33	30.72	25.72	22.39	13.39	—
2010	15.22	16.09↓	23.08	28.66	31.97	35.05	36.11	39.08	34.38↑	29.13	23.22	17.88	27.49
2011	15.89	19.17	25.33↑	31.06↑	31.72	36.83↑	37.72	39.89↑	35.94↑	28.72	23.50	16.56	28.56
2012	19.67↑	19.00	24.56↑	28.50	—	—	—	—	—	—	—	—	—
**Mean min. temp. (°C)**	**4.78**	**6.61**	**10.22**	**14.17**	**18.94**	**22.28**	**23.33**	**23.33**	**20.44**	**15.39**	**10.00**	**5.44**	**14.6**
2009	—	—	—	14.00	20.06	23.61	25.28	24.44	21.17	15.83	10.39	4.17	—
2010	3.66	4.66↓	10.44	16.05↑	19.08	24.08	24.91	25.41	21.69	14.58	10.22	6.72	15.13
2011	4.00	6.00	12.50↑	17.39↑	19.11	23.78	25.11	25.56	21.11	14.61	10.33	6.72	15.50
2012	6.83	9.83	14.50	17.00	—	—	—	—	—	—	—	—	—
**Mean precipitation (mm)**	**80.52**	**69.85**	**81.03**	**64.52**	**114.81**	**110.50**	**55.12**	**68.33**	**84.33**	**124.21**	**78.99**	**79.50**	**1016.30**
2009	—	—	—	155.19↑	35.81↓	0.25↓	61.47↑	17.02↓	188.47↑	206.76↑	87.38	71.37	987.30
2010	75.18	42.91	41.91↓	13.97↓	68.07↓	150.36↑	16.00↓	7.87↓	101.6↑	11.94↓	41.91↓	53.85↓	625.57↓
2011	75.95	15.49↓	17.5↓3	0.00↓	85.60↓	72.90↓	1.02↓	7.37↓	57.15↓	24.13↓	61.21↓	87.12	505.46↓
2012	70.62	236.22↑	219.96↑	14.47↓	—	—	—	—	—	—	—	—	—

Note: The above data are from NOAA Online Weather Data (NOWData) 1981–2010 for College Station, Texas (http://www.nws.noaa.gov/climate/xmacis.php?wfo=hgx, accessed 24 April 2012).

### Modular green roof trays

**Fig. 1:**
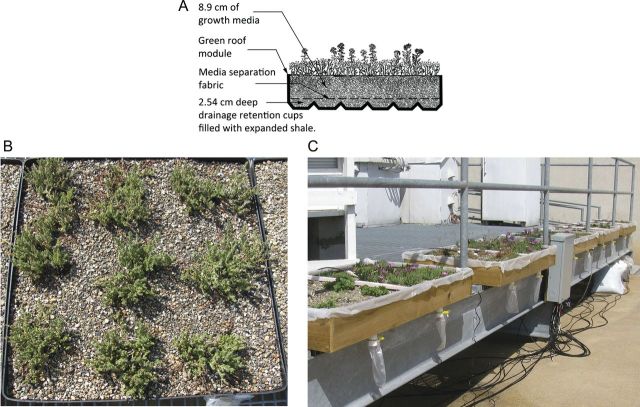
**The configurations of the modular green roof trays are shown in** **a cross-section of materials (A), arrangement of plants in the modules (B) and module replicate placement along the rooftop platform (C)**.

Plants were grown in 0.61 × 0.61 m rigid plastic modular green roof trays (Fig. 1A) (TectaGreen™, Tecta America Corp., Skokie, IL, USA). The modules were 11.4 cm deep (4.5″) with 8.9 cm (3.5″) depth of FLL-compliant growth media (Rooflite^®^drain, Skyland, Avondale, PA, USA) and a 2.54 cm (1″) depth of expanded shale filled inside the drainage retention cups (Fig. 1A). A non-degradable landscape fabric was provided by the green roof vendor and was placed between the two layers of substrate materials to maintain their separate functions. No fertilizer was applied during the investigations. Further details regarding the green roof system used in this study are described in greater detail in Aitkenhead-Peterson *et al.* (2010).

### Plant selection

Our initial list of prospective plants included over 100 species. Several variables were used to narrow down the list, including a plant's reported capacity for drought tolerance or avoidance, cold and heat tolerance, ability to withstand sustained exposure to solar radiation and wind, adaptability to shallow substrates, capacity to reproduce, nativity to the region and plant life-form. Since the green roof substrate we investigated was shallow (8.9 cm) and reliable, and green roof guidelines for Europe (FLL 2008) suggest that forbs and grasses need more than 12.7 cm to thrive on green roofs, we looked to other forms of plants such as succulents and subshrubs. However, one species of grass (*Nassella tenuissima*) was investigated. The 15 species we investigated (Table 2) are a mix of native and exotic shallow-rooted species that exhibit good resistance to drought and heat stress. None was identified as invasive in Texas according to the USDA Plant Finder Database.

**Table 2:** **List of and attributes of plants investigated for use on extensive green roofs in College Station, Texas.** The International Plant Names Index (IPNI) is used for plant nomenclature. Nativity to Texas or the USA means the species is endemic according to the online USDA Plants Database: http://plants.usda.gov/java/. Minimum cold hardiness is the minimum temperature the species is commonly referenced according to the online USDA Cold Hardiness Map. http://planthardiness.ars.usda.gov/PHZMWeb/

Genera	Species	Family	Nativity	Min. cold hardiness	Habit	Life-form
*Bulbine*	*frutescens*	Liliaceae	S. Africa	−6.7 °C	Accent	Succulent
*Delosperma*	*cooperi*	Aizoaceae	S. Africa	−20 °C	Groundcover	Succulent
*Dichondra*	*argentea*	Convolvulaceae	S. USA	−1.7 °C	Groundcover	Forb
*Graptopetalum*	*paraguayense*	Crassulaceae	Mexico	−6.7 °C	Accent	Succulent
*Lampranthus*	*spectabilis*	Aizoaceae	S. Africa	−6.7 °C	Accent	Succulent
*Malephora*	*lutea*	Aizoaceae	S. Africa	−9.4 °C	Groundcover	Succulent
*Manfreda*	*maculosa*	Agavaceae	Texas	−15 °C	Accent	Subshrub/forb
*Myoporum*	*parvifolium*	Myoporaceae	Australia	−3.9 °C	Groundcover	Creeping shrub
*Nassella*	*tenuissima*	Poaceae	Texas	−20 °C	Accent	Graminoid
*Phemeranthus*	*calycinus*	Portulacaceae	Texas	−20 °C	Accent	Succulent
*Sedum*	*kamtschaticum*	Crassulaceae	Asia	−34.4 °C	Mat-forming	Succulent
*Sedum*	*mexicanum*	Crassulaceae	C. America	−9.4 °C	Spreader	Succulent
*Sedum*	*moranense*	Crassulaceae	Mexico	−9.4 °C	Mat-forming	Succulent
*Sedum*	*tetractinum*	Crassulaceae	China	−15 °C	Mat-forming	Succulent
*Stemodia*	*lanata*	Scrophulariaceae	Texas	−9.4 °C	Groundcover	Forb

### Plant installations

The initial investigation ran from 2 April 2009 to 19 October 2010. All plants were removed at the end of the study. Twenty-seven plants were established in three monoculture replicate trays (*n* = 3) for *Delosperma cooperi*, *Sedum kamtschaticum* and *Phemeranthus calycinus* syn. *Talinum calycinum.* Nine 5-cm-deep nursery-grown plant plugs were installed and spaced 20.32 cm apart from each other in rows (Fig. 1B). Nine planted module replicates were placed in a completely randomized arrangement along the edge of the study platform (Fig. 1C).

The second plant installation study began on 10 March 2010 and ended on 19 October 2010. All plants were left in place at the end of plant measurements. Nine additional green roof modules were installed at the research site identical to and adjacent to those used in the first study. New plant species included: *Lampranthus spectabilis* (10 cm spacing), *Malephora lutea* (10 cm spacing) and *Sedum mexicanum* (10 cm spacing). We began to investigate denser plant spacing to increase shading on the growth media and to help retain soil moisture. *Delosperma cooperi*, *Sedum moranense* and *P. calycinus* were also planted but were mixed in trays with three of each species for a total of nine plants in three trays (*n* = 3). Twenty-seven plants of *Bulbine frutescens* and *S. moranense* were studied in monoculture plantings (20.32 cm spacing) with nine plants per three tray replicates (*n* = 3).

The third plant study began on 16 February 2011 with seven additional species including: *Graptopetalum paraguayense*, *Dichondra argentea*, *Stemodia lanata*, *Nassella tenuissima*, *Manfreda maculosa*, *Myoporum parvifolium* and *Sedum tetractinum.* Several species from the previous investigation were also re-examined: *B. frutescens*, *M. lutea*, *S. mexicanum*, *L. spectabilis*, *S. kamtschaticum* and *P. calycinus*. Plants were completely randomized into nine module replicates by three groups: succulents only, herbaceous species, and a mix of succulents and herbaceous species, with three trays per group (*n* = 3). Plants were spaced 5–10 cm apart and were not organized in rows, to achieve a vegetative cover of mixed species.

### Maintenance

Irrigation was applied only during the first several weeks of establishment, and only when natural rainfall subsided to a point where irrigation was determined as beneficial for the establishment of plants. Irrigation was done by hand watering at a rate of 5.3 mm depth of water per module with a sprinkling can once every 7–10 days if there was no rain. For the April 2009 installations, plants were watered 14 times between April and August and no supplemental irrigation was applied after 24 August 2009. The 2010 plant installations were watered by hand on 14 and 29 March. The 2011 plantings received supplemental watering on 18 February, 25 February, 25 March, 5 April, 16 April, 27 April, 10 May and 1 August.

### Plant measurements

Monthly plant growth measurements [growth index (GI)] and photographs were taken for *D. cooperi*, *S. kamtschaticum* and *P. calycinus* in 2009, and *B. frutescens*, *D. cooperi*, *S. moranense*, *S. kamtschaticum* and *P. calycinus* in 2010. A plant GI was devised as a measurement of the volumetric plant canopy area (cm^3^) and porosity of each plant's canopy. This method is a modification to the measurement method initially used by Schroll *et al*. (2009). An idealized sphere was taken of the plant canopy with the longest width by the longest perpendicular width (Schroll *et al*. 2009); however, we also measured the mean canopy height as well. The GI was calculated by multiplying the height of the plant canopy by the two-dimensional area of the plant canopy and the estimated percentage of live growth occupying the area of the canopy. Since plants were intermixed in trays, photographic or quadrant grid methods would not allow measurement of growth for intermixed species as the plants matured. Dead plants were left in place and were not included in the descriptive analyses. Weeds were not pervasive but were removed so that only the studied species were allowed to compete for resources.

For 2011 plant installations, photographs were taken once a month and a plant health rating was calculated at the end of 1 year of growth on 4 April 2012. The visual inspection resulted in plant health ratings based on the following: 1 = severe decline; 2 = some discolouring; 3 = slight distress; 4 = plant is healthy; 5 = healthy and evidence of reproduction. Monthly growth means and standard errors of species cover of modular trays were analysed statistically to determine the growth rates and survival.

Species differences in plant health ratings and GI analyses were analysed using analysis of variance (ANOVA) (Stata 12 software, StataCorp, College Station, TX, USA) using a mean value for each species per replicate tray (usually *n* = 3). Survivorship for five species (*P. calycinus*, *S. kamtschaticum*, *D. cooperi*, *S. moranense* and *B. frutescens*) was analysed using a parametric survival analysis (JMP PRO 10, SAS Institute, Cary, NC, USA). Owing to the very low replication rates for survivorship analysis, differences in survivorship between species were based upon the percentage survivorship of all individuals (27) planted rather than a mean survivorship per tray. Species survival rates were determined for each species by dividing the number of plants that survived by the number of plants.

## Results

Over the three investigations, four species had survival rates of 100 %, including *G. paraguayense*, *M. lutea*, *M. maculosa* and *P. calycinus* (Table 3). Several species had some mortality, including *B. frutescens*, *L. spectabilis*, *N. tenuissima* and *S. kamtschaticum* with maximum survival rates of 44, 56, 22 and 26 %, respectively. Six species had no survivors during the study, including *S. moranense*, *D. cooperi*, *D. argentea*, *S. lanata*, *M. parvifolium* and *S. tetractinum* (Table 3).

**Table 3:** Summary of plants installed (PI) and percentage survival (S) on unirrigated green roofs from April 2009 to April 2012.

Genera	Species	2009 PI, S (%)	2010 PI, S (%)	2011–2012 PI, S (%)
*Phemeranthus*	*calycinus*	27, 100	9, 100	18, 100
*Sedum*	*kamtschaticum*	27, 26	—	12, 0
*Delosperma*	*cooperi*	27, 0	9, 0	—
*Malephora*	*lutea*	—	9, 0	15, 100
*Lampranthus*	*spectabilis*	—	9, 0	27, 56
*Bulbine*	*frutescens*	—	27, 0	36, 44
*Sedum*	*mexicanum*	—	9, 11	36, 8
*Sedum*	*moranense*	—	27, 0	—
*Manfreda*	*maculosa*	—	—	18, 100
*Graptopetalum*	*paraguayense*	—	—	9, 100
*Nassella*	*tenuissima*	—	—	36, 22
*Dichondra*	*argentea*	—	—	9, 0
*Myoporum*	*parvifolium*	—	—	9, 0
*Stemodia*	*lanata*	—	—	9, 0
*Sedum*	*tetractinum*	—	—	12, 0

**Fig. 2:**
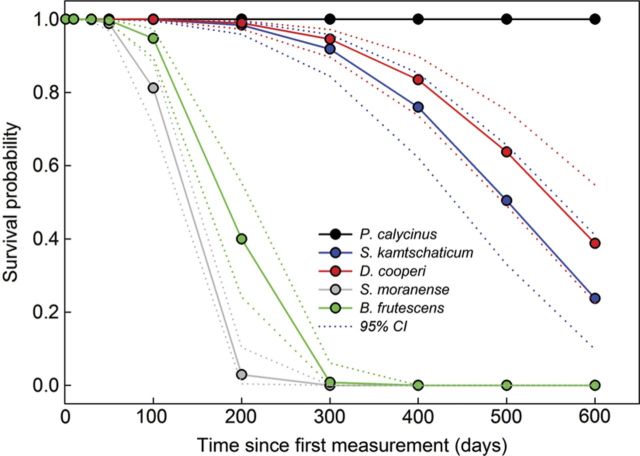
**Modelled survival rate of *S. kamtschaticum*, *D. cooperi*, *B. frutescens and S. moranense* based upon 27 individuals using parametric survival analysis.** Dotted lines indicate 95 % confidence interval. *P* species < 0.001 as determined using a *χ*^2^ test (*χ*^2^ = 149.1). Median survival time for each species is reported in Table 4.

Of the species studied in depth using a parametric survival analysis, *P. calycinus* was the only species with 100 % survival from Day 0 to Day 600 (Fig. 2). Median survival time for *D. cooperi* (655 days) was longer than that for *S. kamtschaticum* (223 days) and *B. frutescens* (191 days), while *S. moranense* (158 days) had a shorter median lifespan than all species except *B. frutescens* (Fig. 2; Table 4).

**Table 4:** Median survival time and 95 % confidence interval (CI) for *B. frutescens*, *D. cooperi*, *S. kamtschaticum* and *S. moranense. Phemeranthus calycinus* was not included in the analysis because no mortality was observed.

Species	Median survival (days)	Lower 95 % CI	Upper 95 % CI
*B. frutescens*	191	170	215
*D. cooperi*	655	582	737
*S. kamtschaticum*	223	195	255
*S. moranense*	158	141	177

**Fig. 3:**
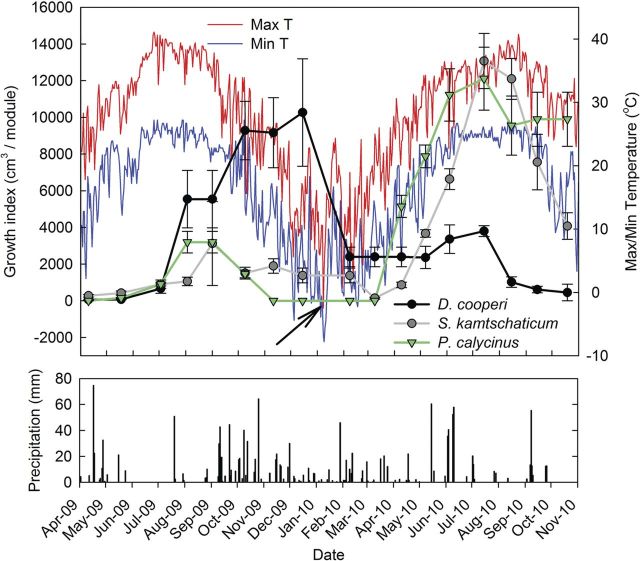
**Graph showing a comparison of species (initial study) monthly GI (cm^3^) means with maximum and minimum air temperatures (°C) and precipitation events (mm).** The arrow points to the climate event when maximum air temperatures did not rise above freezing and the minimum temperature for the day was −7.8 °C.

Plant growth during the 2009 growing season for *D. cooperi* outperformed all other species with a maximum GI of 1131 cm^3^ in December, but quickly declined after cold air temperatures damaged plants and top growth remained minimal and never fully recovered (Fig. 3). *Delosperma cooperi* planted in 2010 faded during the mid-to-late summer drought of 2010. Most top growth had died back and it was assumed that the plants were dead by the end of the experimental measurements in October. All of the 2010 *D. cooperi* were left in place and none of them emerged in 2011; however, top growth emerged during the spring of 2012 but no measurements were taken.

**Fig. 4:**
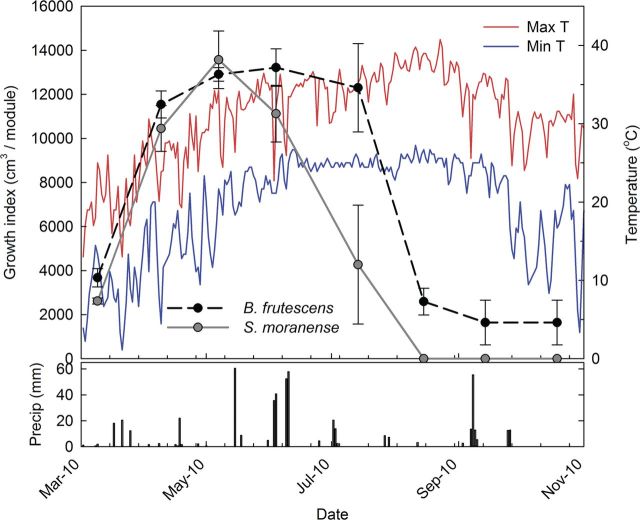
Graph showing a comparison of species (2010 study) monthly GI (cm^3^) means with maximum and minimum air temperatures (°C) and precipitation events (mm).

During July, *S. kamtschaticum* achieved a maximum GI of 1452 cm^3^, which was a greater volume than for any other species and maintained dominance until August (Fig. 3). The species *P. calycinus* performed consistently throughout the hottest and driest periods; however, its dormancy cycle begins in early fall, thus its GI of zero from November 2009 to March 2010 and October 2010 was due to dormancy (Fig. 3). During April 2010, the GI for *B. frutescens* was the highest of all species at 1507 cm^3^, but plant growth began to decline after maximum daytime air temperatures were consistently over 37.0 °C and dry conditions persisted (Fig. 4). The GI for *S. moranense* modules peaked at 1468 cm^3^ in June, but declined quickly thereafter and all the plants were dead after 150 days (Figs 2 and 4).

Four species of the 2011 installations survived without any losses, including *G. paraguayense*, *P. calycinus*, *M. maculosa* and *M. lutea* (Table 3). Several species suffered some mortality, including *B. frutescens*, *N. tenuissima*, *L. spectabilis*, and *S. mexicanum* (Table 3). There were several species with no surviving plant replicates, including *D. argentea*, *S. lanata*, *M. parvifolium*, *S. tetractinum* and *S. kamtschaticum* (Table 3). The healthiest species included *M. lutea*, *M. maculosa* and *P. calycinus* with mean health ratings of 4.0, *L. spectabilis* had a mean rating of 3.7 and *G. paraguayense* had a mean health rating of 3.5 (Table 5).

**Table 5:** **Results of one-way ANOVA comparison (Bonferroni) of mean health ratings (measured on 4 April 2012) testing the significance between species.** The difference between species health rating means is shown, and those that are statistically significant (*P* < 0.05) are highlighted in bold. Plant species health rating key: 0 = no survivors; 1 = severe decline; 2 = some discolouring; 3 = slight distress; 4 = plant is healthy; 5 = healthy and evidence of reproduction.

	*B. frutescens*	*G. paraguayense*	*N. tenuissima*	*S. mexicanum*	*M. lutea*	*M. maculosa*	*L. spectabilis*	*P. calycinus*
Mean health rating	3.667	3.500	2.929	2.333	4.000	4.000	3.714	4.000
Standard deviation	0.516	0.000	0.886	0.577	0.000	0.000	0.469	0.000
*G. paraguayense*	−0.167							
*N. tenuissima*	**−0.738**	−0.571						
*S. mexicanum*	**−1.333**	**−1.167**	−0.595					
*M. lutea*	0.333	**0.500**	**1.071**	**1.667**				
*M. maculosa*	0.333	**0.500**	**1.071**	**1.667**	0.000			
*L. spectabilis*	0.048	0.214	**0.786**	**1.381**	−0.286	−0.286		
*P. calycinus*	0.333	**0.500**	**1.071**	**1.667**	0.000	0.000	0.286	

## Discussion

Our findings indicate that there are several species that performed well in south-central Texas with minimal watering during establishment and no watering thereafter even though area climate conditions were warmer and drier than long-term means, especially during 2011 when College Station was under exceptional drought conditions from 5 April 2011 to 28 March 2012 (Nielsen-Gammon 2011). On several green roofs in South Florida (tropical climate) establishment of plants on shallow green roofs (14 cm deep) without irrigation was tested and it was recommended that a minimum depth of 15 cm was needed to support plant growth (Livingston *et al*. 2004). Our plant establishment findings are the first report of species establishing on very shallow (<12 cm deep) green roofs in a humid subtropical climate with minimal irrigation during establishment and termination of irrigation thereafter. Our results demonstrate that it is possible to find plant species that can survive and even thrive on very shallow unirrigated green roofs in warm subtropical climates.

The top-performing species for survival included *P. calycinus*, *G. paraguayense*, *M. lutea* and *M. maculosa*. Only one species, *P. calycinus*, was found re-seeding onto other nearby green roof trays. This can be a desirable trait for green roofs, especially if the green roof is dominated by plant species that spread only by rhizomes or shoots, as long as the species is not aggressive or invasive in the landscape. Several species had decent survival rates and appeared to suffer from cold temperatures during their establishment year. *Malephora lutea* exhibited complete mortality during the winter of 2010, when an unusually long period (>24 h) of below freezing temperatures occurred (Fig. 4), but no mortality for those installed in 2011. All of the *L. spectabilis* died during 2010, but there was 44 % survival during the winter of 2011. The persistence of those two species through April 2012 demonstrates that they can establish when winter conditions are not abnormally cold (Table 1).

*Delosperma cooperi* showed a surprising capacity to survive even though top growth had ceased to exist. Plants suffered total canopy dieback in 2010, and failed to produce top growth during the following year, but re-emerged after consistent rainfalls returned to the region in late 2011 and early 2012. Our findings reinforce those by Rowe *et al*. (2012), where they also found changes in plant species success over multi-year periods.

*Dichondra argentea*, *S. lanata*, *M. parvifolium*, *S. tetractinum* and *S. kamtschaticum* failed to establish in 2011, which was probably due to record drought and heat conditions during the spring and summer of 2011. Several *S. kamtschaticum* had successfully established during the 2009 experiment; however, irrigation was applied during 2009 in early summer. In 2011, plants were installed 2 months earlier than in 2009 and irrigation was stopped earlier. The establishment period with irrigation was apparently not long enough to help *S. kamtschaticum* survive the extreme dry 2011 summer without supplemental irrigation beyond May. College Station did not experience unusually high levels of night-time humidity throughout the 2011 growing season compared with the 2009/2010 growing seasons, and therefore the high mortality of *S. kamtschaticum* is probably not due to high night-time humidity. We suggest that *S. kamtschaticum*, *D. argentea*, *S. lanata* and *S. tetractinum* are still worthy of further study during more normal climate conditions or with deeper substrates, or perhaps with consistent irrigation throughout the entire first growing season. These species are drought and heat tolerant, but they have demonstrated difficulty establishing with limited irrigation under extreme drought and high-temperature conditions.

All of the species with 100 % survival were succulents. One gramminoid species was investigated (*N. tenuissima*) and it had a 22 % survival rate (Table 3). In terms of plant form, all of the top performers were erect plants except *M. lutea*. It is possible that the vertical stature favours survival in high-light environments by minimizing interception of solar radiation and thus reducing heat load and potential transpiration rates.

Since representatives of both native and non-native species had high survival, adaptability of plant species to the conditions of the substrate and microclimate (high light, wind exposure, local precipitation patterns) is perhaps a more important predictor of success than nativity of the species to the region (Durhman *et al*. 2006, 2007). From an ecological perspective, however, it would be better to make use of native plants on green roofs where possible, to provide habitat for native resident and migrating wildlife and insects. Testing plants for green roofs that also provide for local or migrating wildlife is plausible and could help conserve biodiversity with green roof vegetation (Kowarik 2011).

## Conclusions and forward look

In summary, four out of 15 species planted in 11.4-cm-deep modular green roof trays—*G. paraguayense*, *M. lutea*, *M. maculosa* and *P. calycinus*—survived without losses. Six species—*B. frutescens*, *D. cooperi*, *L. spectabilis*, *N. tenuissima*, *S. kamtschaticum* and *S. mexicanum*—had varied performance. Five species—*D. argentea*, *S. lanata*, *M. parvifolium*, *S. moranense* and *S. tetractinum*—had no survivors. It is possible that the species with varied performance may perform better if provided with irrigation or deeper substrates. The outcomes of this study demonstrate that there may be several plant species for use on shallow unirrigated green roofs in humid subtropical climates, showing that green roofs are a viable alternative roofing type in spite of the more challenging climatic conditions.

## Sources of funding

Funding for this study was provided by Texas A&M University, College of Architecture Research and Interdisciplinary Council Grant Program (CRIC); materials were donated by TectaAmerica™, Rooflite™, Emory Knoll Nursery and Joss Growers Nursery.

## Contributions by the authors

All the authors contributed to a similar extent overall.

## Conflict of interest statement

None declared.

## References

[PLS049C26] Aitkenhead-Peterson J, Dvorak B, Volder A, Stanley N (2010). Chemistry of growth medium and leachate from green roof systems in south-central Texas. Urban Ecosystems.

[PLS049C1] Barker JK, Lubell DJ (2012). Effects of species proportions and fertility on *Sedum* green roof modules. HortTechnology.

[PLS049C2] Cantor S (2008). Green roofs in sustainable landscape design.

[PLS049C3] Dunnett N, Kingsbury N (2004). Planting green roofs and living walls.

[PLS049C4] Dunnett N, Nagase A (2007). The dynamics and visual impact of planted and colonizing species on a green roof over 6 growing seasons 2001–2006: influence of substrate depth.

[PLS049C5] Durhman AK, Rowe DB, Rugh CL (2006). Effect of watering regimen on chlorophyll fluorescence and growth of selected green roof plant taxa. HortScience.

[PLS049C6] Durhman AK, Rowe DB, Rugh CL (2007). Effect of substrate depth on initial growth, coverage, and survival of 25 succulent green roof plant taxa. HortScience.

[PLS049C7] Dvorak B, Volder A (2010). Green roof vegetation for North American ecoregions: a literature review. Landscape and Urban Planning.

[PLS049C8] **Earth Pledge** (2005). Green roofs ecological design and construction.

[PLS049C9] **FLL** (2008). Guidelines for the planning, construction and maintenance of green roofing.

[PLS049C10] Hauth E, Liptan T (2003). Plant survival findings in the Pacific Northwest.

[PLS049C11] Köehler M (2003). Plant survival research and biodiversity: lessons from Europe.

[PLS049C12] Köehler M (2007). Long-term vegetation research on two extensive green roofs in Berlin. Urban Habitats.

[PLS049C13] Kottek M, Grieser J, Beck C, Rudolf B, Rubel F (2006). World map of the Koppen–Geiger climate classification updated. Meteorologische Zeitschrift.

[PLS049C14] Kowarik I (2011). Novel urban ecosystems, biodiversity, and conservation. Environmental Pollution.

[PLS049C15] Larkin JT, Bomar WG (1983). Climatic atlas of Texas.

[PLS049C16] Liu TC, Shyu GS, Fang WT, Liu SY, Cheng BY (2012). Drought tolerance and thermal effect measurements for plants suitable for extensive green roof planting in humid subtropical climates. Energy and Buildings.

[PLS049C17] Livingston EH, Miller C, Lohr M (2004). Green roof design and implementation in Florida.

[PLS049C18] Lundholm J, MacIvor JS, MacDougall Z, Ranalli M (2010). Plant species and functional group combinations affect green roof ecosystem functions. PLoS ONE.

[PLS049C19] MacIvor JS, Lundholm J (2011). Performance evaluation of native plants suited to extensive green roof conditions in a maritime climate. Ecological Engineering.

[PLS049C20] Nielsen-Gammon J (2011). The 2011 Texas drought: a briefing packet for the Texas legislature. 31.

[PLS049C21] Rowe DB, Getter KL, Durhman AK (2012). Effect of green roof media depth on Crassulacean plant succession over seven years. Landscape and Urban Planning.

[PLS049C22] Schroll E, Lambrinos J, Sandrock D (2009). Irrigation requirements and plant survival on northwest green roofs.

[PLS049C23] Snodgrass E, Snodgrass L (2006). Green roof plants.

[PLS049C24] Sutton RK, Harrington JA, Skabelund L, MacDonagh P, Coffman RR, Koch G (2012). Prairie-based green roofs: literature, templates, and analogs. Journal of Green Building.

[PLS049C25] Williams NSG, Rayner JP, Raynor KJ (2010). Green roofs for a wide brown land: opportunities and barriers for rooftop greening in Australia. Urban Forestry & Urban Greening.

